# *Ganoderma lucidum* Modulates Glucose, Lipid Peroxidation and Hepatic Metabolism in Streptozotocin-Induced Diabetic Pregnant Rats

**DOI:** 10.3390/antiox11061035

**Published:** 2022-05-24

**Authors:** Fabia Judice Marques Viroel, Leticia Favara Laurino, Érika Leão Ajala Caetano, Angela Faustino Jozala, Sara Rosicler Vieira Spim, Thaisa Borim Pickler, Michelle Klein Sercundes, Marcela C. Gomes, Alessandre Hataka, Denise Grotto, Marli Gerenutti

**Affiliations:** 1 Department of Pharmacy, University of Sorocaba, Sorocaba 18023-000, Brazil; fabia.viroel@prof.uniso.br (F.J.M.V.); leticiafavaralaurino@gmail.com (L.F.L.); erikacaetano01@hotmail.com (É.L.A.C.); angela.jozala@prof.uniso.br (A.F.J.); sara.spim@uniso.br (S.R.V.S.); thaisa.pickler@uniso.br (T.B.P.); michellesercundes@gmail.com (M.K.S.); 2 Department of Veterinary Clinical Sciences, School of Veterinary Medicine and Animal Science, São Paulo State University (UNESP), Botucatu 18610-307, Brazil; marcelacgomes92@gmail.com (M.C.G.); hataka@fmvz.unesp.br (A.H.); 3 Departament of Biomaterials and Regenerative Medicine, School of Medicine, Pontifical Catholic University of São Paulo–PUC SP, Sorocaba 18030-070, Brazil; marli.gerenutti@gmail.com

**Keywords:** Gestational Diabetes Mellitus, *Ganoderma lucidum*, oxidative stress, glucose, rat

## Abstract

The consumption of functional foods, such as mushrooms, apparently influences Gestational Diabetes Mellitus (GDM), and brings benefits to maternal-fetal health. *Ganoderma lucidum* contains a variety of bioactive compounds, such as polysaccharides, proteins and polyphenols that are able to control blood glucose and be used in anti-cancer therapy. We aimed to evaluate the effects of the consumption of *Ganoderma lucidum* (Gl) on maternal-fetal outcomes in streptozotocin-induced GDM (GDM-STZ). Pregnant rats were exposed to Gl (100 mg/kg/day) before and after the induction of GDM-STZ (single dose 40 mg/kg) on the eighth pregnancy day. Biochemical and oxidative stress parameters, reproductive performance and morphometry of fetuses were assessed. Gl reduced the glycemic response in the oral glucose tolerance test. Moreover, Gl decreased AST and ALT activities. GDM increased lipid peroxidation, which was reverted by Gl. Catalase and glutathione peroxidase activities were decreased in GDM and the administered Gl after the fetus implantation increased catalase activity. Measurements of the fetal head, thorax, craniocaudal and tail showed greater values in fetuses from rats exposed to Gl compared to GDM. *Ganoderma lucidum* has an encouraging nutritional and medicinal potential against GDM, since it modifies glucose metabolism, reduces lipid peroxidation, and has protective effects in fetuses born from GDM dams.

## 1. Introduction

*Ganoderma lucidum* is a medicinal mushroom for treating assorted diseases. It has a variety of bioactive compounds, such as polysaccharides, proteins, terpenoids, sterols, and polyphenols [1,2]. Its first scientific registration was in 1934, and since then *Ganoderma lucidum* has been called the “mushroom of immortality”, known as “Lingzhi in China, and Reishi in Japan” [3,4]. Nowadays, *Ganoderma lucidum* is consumed as a nutraceutical in the form of soup, tea, or other mixtures by predominantly Asian populations as a way to improve well-being and health [5].

*Ganoderma lucidum* is the most famous medicinal mushroom in the Chinese pharmacopeia and has been extensively researched for its medical and clinical applications [6]. Chen et al. demonstrated that compounds from the *Ganoderma lucidum* fruit body could be used as anti-cancer agents and for controlling blood glucose [7].

Diabetes Mellitus (DM) is a chronic, degenerative endocrine disease characterized by the decreased secretion of insulin by the pancreas (type 1 DM) or increased insulin resistance by the body’s cells (type 2 DM), increasing blood glucose levels. Both types are complex and serious, and they can trigger oxidative stress and chronic-degenerative changes, such as nephropathy, retinopathy, neuropathies, and cardiovascular and cerebrovascular alterations. The prevalence of DM has been dramatically increasing worldwide, and it is projected to continue at the same rate if proper measures are not taken [8,9,10].

Gestational Diabetes Mellitus (GDM) is a global public health problem. The pathophysiology of GDM is not fully understood but it has been linked to hormonal imbalance, affecting insulin sensitivity and pancreatic β-cell dysfunction. If GDM is not properly treated, hyperglycemia may damage endothelial cells, resulting in vascular dysfunction associated with hypertension. This state has a higher risk of premature rupture of the placenta and premature delivery, fetal pelvic presentation and macrosomic fetus [11]. Conditions, such as hypoxia, infection and diabetes during pregnancy are associated with changes in placental and trophoblast cells that affect the intrauterine environment and disrupt normal fetal development. Neonatal complications include asphyxia, hypoglycemia, jaundice, bacterial infections, respiratory distress syndrome and birth trauma [12].

In this context, monitoring pregnant women with GDM is very important, and metabolic and antioxidant control through adjustments in diet and exercise should be included. Insulin is used when lifestyle modifications are not sufficient to gain glycemic control. Nutritional therapies, such as high-fiber and antioxidants, and low-glycemic-index diets, have been shown to improve insulin sensitivity and glucose tolerance, which may reduce the risk for GDM [13,14,15].

The great challenge of this study was to investigate the effects of *Ganoderma lucidum* on maternal glucose metabolism, biochemical parameters and oxidative stress, and fetal development parameters in pregnant rats with streptozotocin (STZ)-induced GDM.

## 2. Materials and Methods

### 2.1. Sample Preparation and Chromatographic Fingerprint Analysis

Fresh *Ganoderma lucidum* (Gl) mushrooms were lyophilized until a 10% dry mass was obtained. The dried sample was milled and sieved to a homogeneous powder.

Phenolic compounds and glucans are the main active components of Gl. The quantification of phenolic compounds was carried out by colorimetric analysis, in triplicate, according to Ramirez-Anguiano et al. [16]. The results were expressed in milligrams of gallic acid equivalent (GAE)/mg of sample on a dry basis. The quantification of glucans was carried out in triplicate as well, by enzymatic and chemical hydrolysis, following the commercial yeast beta-glucan kit protocol (Megazyme^®^, Lansing, MI, USA).

Moreover, the specific composition fingerprint of the Gl was carried out using High Performance Liquid Chromatography (HPLC). For this, 2.0 g of dried Gl were extracted with 40 mL CHCl_3_ in an ultrasonic water bath for 20 min, according to Yang et al. [17]. The extracted solution was evaporated at 35 °C to dryness in a rotary evaporator (Büchi^®^ R-215 Rotavaporation System, V-700 Vacuum Pump and F-108 Chiller). An aliquot of the sample in chloroform was collected before complete drying. Moreover, the remaining dry extract was dissolved in 4.5 mL methanol and filtrated through a 0.45 m membrane filter unit. Then 30 L of each sample solution was analyzed by HPLC.

The fingerprint was performed on a Shimadzu-model Class-VP-HPLC instrument. The methodology followed Yang et al. [17], with some modifications: a C18 column (L 25 cm × 4.60 mm, 5 μm particle size; Thermo Fisher Scientific, Waltham, MA, USA) and 30 µL of injection. The mobile phase consisted of acetonitrile (CH_3_CN) and water containing 0.2% (*v*/*v*) CH_3_COOH. The flow rate was 1.0 mL/min, with column temperature at 35 °C, and detection at 252 nm.

### 2.2. Experimental Design

Gl powder was resuspended in water for oral administration to animals. The daily dose of 100 mg/kg of mushroom was selected based on previous studies with other culinary-medicinal mushrooms [18,19] and on the recommended daily dose of *Ganoderma lucidum* as a dietary supplement by Pharmacopoeia of China [20]. The rats received mushrooms via gavage in two different periods. The GDM+Glb group received it from the 1st to the 19th day of pregnancy and the GDM+Gla group received it from the 9th to the 19th day of pregnancy.

The study was approved by the Animal Ethics Commission of the University of Sorocaba Protocol n° 089/2016. Wistar rats (males, weighing between 250 and 300 g, and females weighing between 180 and 200 g) were maintained in microenvironment isolation cages under standard environmental conditions (23 ± 3 °C and 12:12 h dark/light cycle) and they received food and water ad libitum.

For mating, one male was housed with two females, for a nocturnal period of 12 h and the indication of pregnancy was the presence of spermatozoa in the smear from the vaginal lavage. After confirmation of pregnancy, female rats were randomly and individually allocated into four groups of six animals/group: (I) saline control (SC), (II) GDM saline (GDM+S), (III) GDM+Glb and (IV) GDM+Gla.

### 2.3. Induction of GDM and Oral Glucose Tolerance Test (OGTT)

For the induction of DM, the pregnant rats received an intravenous (caudal vein) injection of streptozotocin (STZ) diluted in citrate buffer (pH 4.5), at a dose of 40 mg/kg, on the eighth day of gestation. The SC animals received the equivalent amount of citrate buffer. After 48 h of STZ administration, the peripheral blood of the auricle was collected for glucose dosing. Animals with glycemia above 120 mg/dL were considered diabetic.

On the 17th day of pregnancy, the OGTT was performed on all pregnant rats. After 6-h fasting, a solution of dextrose (2 g/kg body weight) was administered via gavage, and the blood of the auricle was collected at 0, 10, 20, 30, 60 and 120 min for the determination of glucose levels.

### 2.4. Reproductive Performance and Blood Collection

On the 20th day of pregnancy, females were anesthetized with ketamine (100 mg/kg) and xylazine hydrochloride (6 mg/kg) intraperitoneally. The cesarean procedure was performed with a longitudinal incision in the linea alba to expose the uteruses and ovaries. The maternal blood was collected by venous-hepatic puncture and transferred into two tubes: tube 1, containing anticoagulant ethylenediaminetetraacetic acid (EDTA), for the redox parameters and tube 2, without anticoagulant, for biochemical evaluations.

After that, females were euthanized, and uteruses and ovaries were removed. The uteruses were inspected for the number of fetuses, implantations and resorptions visible to evaluate the post-implantation losses of the embryo and ovaries were sectioned for withdrawal and count of the number of corpus luteum.

### 2.5. Maternal Biochemical Parameters and Redox Status

The biochemical parameters alanine aminotransferase (ALT), aspartate aminotransferase (AST), albumin, lipase, creatinine, urea, glucose, total cholesterol, high density lipoprotein (HDL) and triglycerides were analyzed using Cobas C111, Roche^®^, Basel, Swiss. automatic equipment, following commercial kits. The plasma insulin dosage was performed by the Elisa solid phase test, based on the Sandwich principle, using a commercial rat-specific kit Rat/Mouse Insulin Elisa Kit-Merck^®^ (Sigma-Aldrich, St. Louis, MI, USA).

Total thiols were quantified by the concentration of reduced glutathione (GSH), based on the Ellman method [21]. For this, 150 µL of the blood was vortexed with 100 µL 10% Triton X-100 and 100 µL 30% Trichloroacetic acid (TCA). Then, the sample was centrifuged for 10 min at 4000 rpm. In a cuvette, 900 µL of 1M potassium phosphate buffer (PPB), 50 µL of supernatant, and 50 µL of 10 mM 5,5′-dithio-bis (2-nitrobenzoic acid (DTNB) were mixed, forming a yellow complex. The reading was carried out in a spectrophotometer at 412 nm. A calibration curve with different concentrations of GSH standard was used to calculate GSH concentrations in blood, which were expressed in millimoles per liter of whole blood.

The glutathione peroxidase (GPx) activity in whole blood was determined based on the oxidation of NADPH; 10 µL of whole blood was diluted in 390 µL of 100 mmol/L PPB. A 20 µL aliquot was added to 880 µL of a solution containing GSH, GSH reductase, NADPH, sodium azide, and 100 µL of H_2_O_2_. The GPx activity was monitored for two minutes at 340 nm, according to Paglia and Valentine (1967) [22]. The decrease in absorbance was proportional to the NADPH consumption by the GPx. Enzyme activity was expressed in millimoles (mmol) of NADPH per gram of Hemoglobin, per minute, using Hemoglobin concentration (in grams per liter) for the calculation.

To evaluate the activity of the catalase (CAT) enzyme, the method of Aebi [23] was used, based on the decomposition of H_2_O_2_ by catalase, monitored at 240 nm. For this, the whole blood was diluted at 1:60 in 50 mM PPB. An aliquot of 20 μL was added to 1910 μL of the same PPB as well as 70 μL of H_2_O_2_, initiating the reaction, which was read for three minutes. A constant of variation (κ), related to Hb, per minute, helped in the expression of the catalase enzyme (κ/gHg/min).

Thiobarbituric acid reactive substances (TBARS) were used as a biomarker of lipid peroxidation, according to Ohkawa et al. [24]. Plasma aliquots (150 µL) were mixed with 50 µL sodium hydroxide and 50 µL Milli-Q ultrapure water, and incubated at 60 °C for 30 min, in a shaker. Aliquots of 250 µL of 6% phosphoric acid, 250 µL of 0.8% thiobarbituric acid (TBA) and 100 µL of 10% sodium dodecyl sulfate were added to the samples and put into the 90 °C bath for one hour. Lipid peroxides reacted with TBA in an acidic medium to form a pink compound and were read in a spectrophotometer at 532 nm. A calibration curve with different concentrations of malondialdehyde standard (the main product from lipid peroxidation) was used to calculate TBARS concentrations in plasma, which were expressed in micromoles per liter.

### 2.6. Embryofetal Development

Fetuses were separated from placentas and weighed. Fetuses were measured-cranium, laterolateral of the cranium, anteroposterior of the thorax, laterolateral of the thorax, cranium-caudal and tail-with the aid of a digital caliper. Fetuses were then euthanized with halothane (cell saturation) and fixed in buffered formalin. Subsequently, the brain and kidneys were removed, treated, and stained by hematoxylin and eosin techniques [25] and the slides were analyzed blindly. Transverse sections were performed in the mid-hippocampus region and measurements were taken of the hippocampus and the cerebral cortex. The longitudinal sections of both kidneys were quantitatively analyzed by counting the glomeruli present in each organ.

### 2.7. Statistical Analysis

A level of significance between 1% and 5% was used. The Bartlet test was used to evaluate the homoscedasticity of data. ANOVA was used for the parametric data, followed by the Tukey–Kramer test for multiple comparisons in all experiments. The Chi square test was used for the results in percentage. All data were evaluated in GraphPad Prism version 6.0 (San Diego, CA, USA).

## 3. Results

Regarding active components of Gl, 30 g of β-glucans and 1.8 mg EAG of phenolic compounds/g of dry mushroom were measured. Regarding HPLC fingerprint (Figures S1 and S2), compounds from Gl were identified by comparing the retention time of the samples to standards from another study. Methanolic extract presented eleven peaks comparable to the results found by Yang et al. [17]. The main compounds were Ganoderic acid G, B, AM1, A, D, F, 7,15-dihydroxy-4,4,14-trimethyl-3,11-dioxochol-8-en–24-oic acid, 12-hydroxyganoderic acid D, Ganoderenic acid D, Lucidenic acid D and Ganolucidic acid D. Moreover, examining the Gl sample in chloroform, a novel peak is observed-12-Hydroxyganoderic acid C2–and some of the others are repeated. These triterpenoids are the active compounds commonly found in the fruiting bodies, cultured mycelia, and spores from *G. lucidum* [26,27].

**Figure 1:**
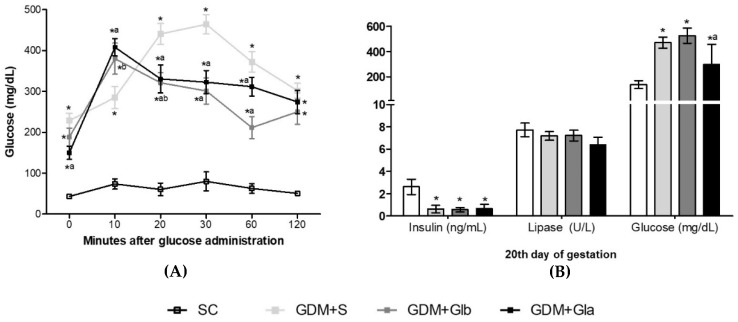
(**A**,**B**) The evolution of GDM-STZ. SC (0.9% saline solution), GDM+S (Diabetic + saline solution 0.9%), GDM+Glb (Diabetic + 100 mg/kg/day of *Ganoderma lucidum* from gestation day 1 to 19), GDM+Gla (Diabetic + 100 mg/kg/day of *Ganoderma lucidum* from gestation day 9 to 19). Data are presented as mean ± SD (*n* = 6). (*) *p* < 0.05 in comparison to the SC group, (a) in comparison to the GDM+S group, (b) in comparison to the GDM+Glb group; One-way ANOVA, followed by Tukey–Kramer’s test.

Figure 1A shows the OGTT curve performed on the 17th day of gestation. The three GDM groups presented high glycemic rates at all times compared to the control. At 20, 30 and 60 min, rats exposed to Gl showed lower glycemic levels than the DGM+S group (F = 19.49; *p* < 0.001). In Figure 1B, all GDM groups had the insulin levels decreased (F = 28.09, *p* < 0.0001) and the glucose levels increased (F = 14.21, *p* < 0.0001) when compared to control. GDM+Gla presented a reduction in glycemia in comparison to GDM+S. No differences were observed in lipase.

**Figure 2:**
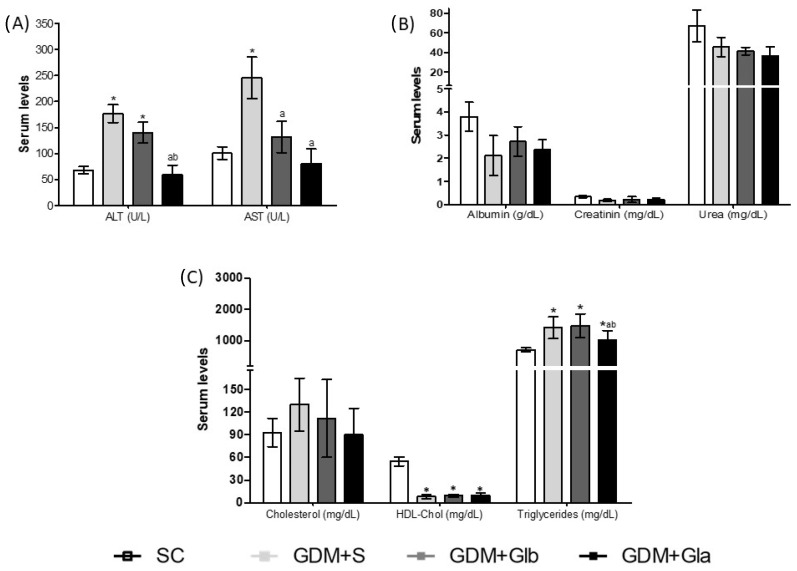
Biochemical parameters in GDM-STZ of maternal serum. SC (0.9% saline solution), GDM+S (Diabetic + saline solution 0.9%), GDM+Glb (Diabetic + 100 mg/kg/day of *Ganoderma lucidum* from gestation day 1 to 19), GDM+Gla (Diabetic + 100 mg/kg/day of *Ganoderma lucidum* from gestation day 9 to 19). (**A**) represent hepatic profile AST (aminotransferase de aspartate), ALT (aminotransferase de alanine). (**B**) shows renal function Albumin, Creatinin and Urea. (**C**) is lipid profile Cholesterol, HDL-Chol (high-density lipoproteins) and Triglycerides. Data are presented as mean ± SD (*n* = 6). (*) *p* < 0.05 in comparison to the SC group, (a) in comparison to the GDM+S group, (b) in comparison to the GDM+Glb group, One-way ANOVA, followed by Tukey–Kramer’s test.

The maternal biochemical profile is shown in Figure 2. Figure 2A shows the hepatic profile, and ALT activity increased in GDM+S and GDM+Glb in relation to SC and GDM+Gla (F = 30.22; *p* < 0.0001). AST activity increased in GDM+S compared to SC and both groups were exposed to Gl (F = 6.884; *p* = 0.0051). Renal function is presented in Figure 2B, and no differences were reported. Regarding the lipid profile (Figure 2C), the triglyceride levels were higher in all GDM groups in relation to SC (F = 25.88; *p* = 0.0025). Despite this rise, the GDM+Gla group presented lower triglycerides when compared to the other diabetics. A significant decrease was observed in HDL-Chol levels in all diabetic groups.

**Figure 3:**
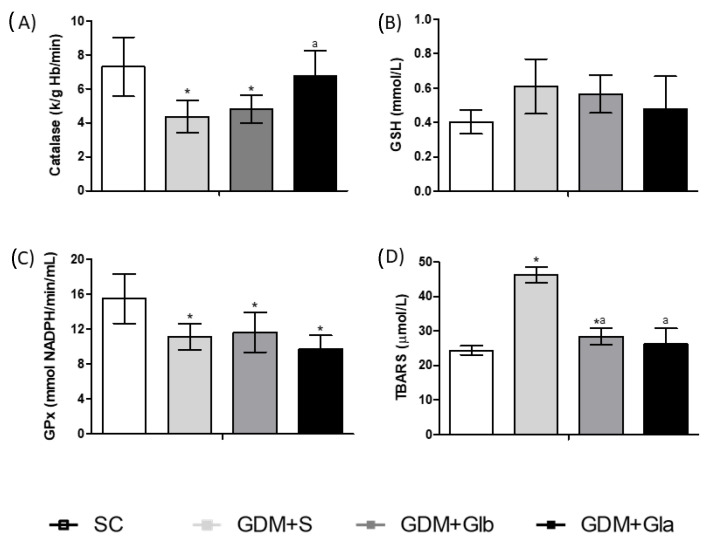
Oxidative stress of pregnant rats. GDM-STZ treated with *Ganoderma lucidum*. SC (0.9% saline solution), GDM+S (Diabetic+saline solution 0.9%), GDM+Glb (Diabetic + 100 mg/kg/day of *Ganoderma lucidum* from gestation day 1 to 19), GDM+Gla (Diabetic + 100 mg/kg/day of *Ganoderma lucidum* from gestation day 9 to 19). (**A**). Catalase (CAT), (**B**). reduced glutathione (GSH), and (**C**). glutathione peroxidase reduction (GPX), (**D**). Thiobarbituric acid reactive substances (TBARS). Data are presented as mean ± SD (*n* = 6). (*) *p* < 0.05 in comparison to SC group, (a) in comparison to the GDM+S group. One-way ANOVA, followed by Tukey–Kramer’s test.

Figure 3 shows the biomarkers of the redox state in maternal blood. CAT activity in GDM+S and GDM+Glb in relation to SC and GDM+Gla tended to decrease (F = 2.733; *p* = 0.0901). In relation to GPx, the GDM groups showed a decrease in enzyme activity. The GDM+S group showed an increased concentration of TBARS when compared to the SC group and to the groups exposed to Gl (F = 16.54, *p* = < 0.0001).

Changes in the reproductive capacity are presented in Table 1. The weight of uteruses and ovaries diminished in GDM+S in relation to SC. The highest percentages of post-implantation losses were observed in all GDM groups in relation to SC.

**Figure 4:**
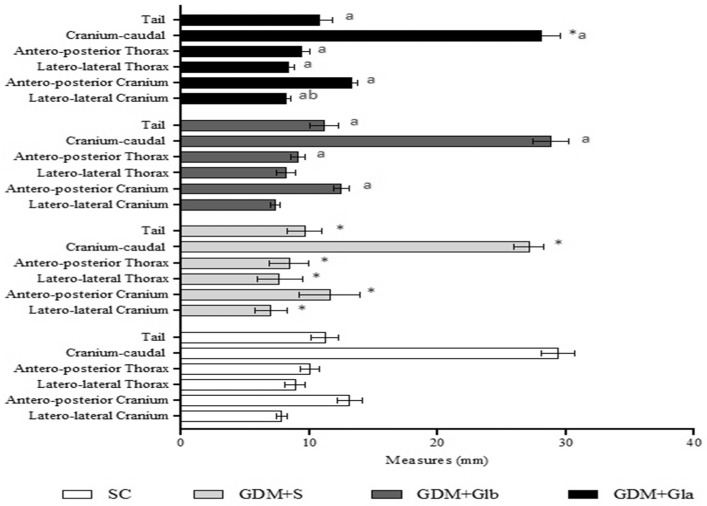
Fetal measurements. GDM-STZ treated with *Ganoderma lucidum*. SC (0.9% saline solution), GDM+S (Diabetic+saline solution 0.9%), GDM+Glb (Diabetic + 100 mg/kg/day of *Ganoderma lucidum* from gestation day 1 to 19), GDM+Gla (Diabetic + 100 mg/kg/day of *Ganoderma lucidum* from gestation day 9 to 19). Data are presented as mean ± SD (*n* = 8). (*) *p* < 0.05 in comparison to SC group, (a) in comparison to the GDM+S group, (b) in comparison to the GDM+Glb group; One-way ANOVA, followed by Tukey–Kramer’s test.

Figure 4 shows the morphometric analyses of fetuses. GDM+S presented a reduction in all parameters in comparison to SC, GDM+Glb and GDM+Gla. GDM+Gla showed a greater laterolateral measurement of the skull when compared to GDM+Glb (F = 24.73; *p* < 0.001).

**Figure 5:**
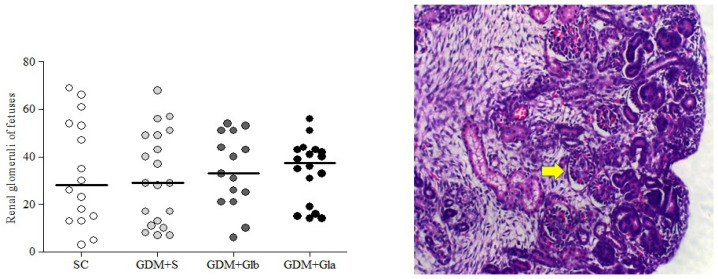
Fetal Glomerulus Count. GDM-STZ treated with *Ganoderma lucidum*. SC (0.9% saline solution), GDM+S (Diabetic+saline solution 0.9%), GDM+Glb (Diabetic + 100 mg/kg/day of *Ganoderma lucidum* from gestation day 1 to 19), GDM+Gla (Diabetic + 100 mg/kg/day of *Ganoderma lucidum* from gestation day 9 to 19). Data are presented as median and dispersion (*n* = 16). *p* > 0.05 in comparison to SC group, One-way ANOVA, followed by Tukey–Kramer’s test. H&E 40X photomicrography in a kidney from a fetus (note: the yellow arrow indicates a glomerulus).

Figure 5 illustrates the median and dispersion of the number of glomeruli of both kidneys from fetuses. No differences among the groups were observed (F = 0.092 and *p* > 0.05).

**Figure 6:**
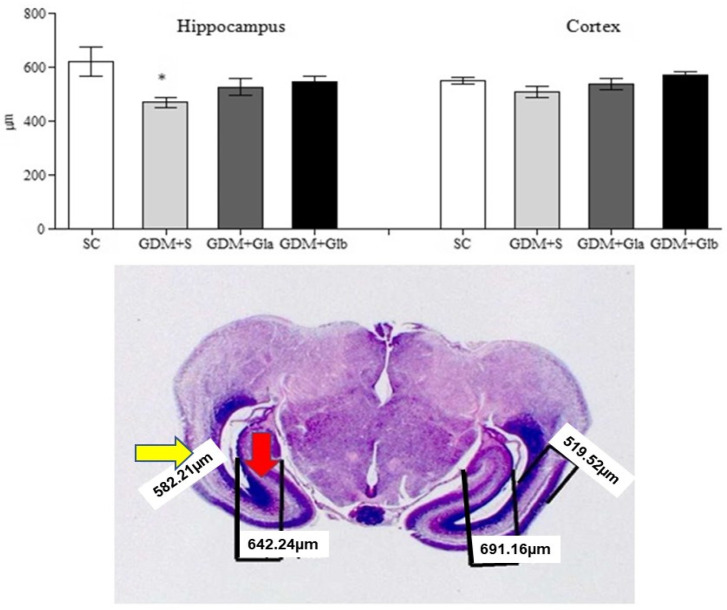
Measurement of the hippocampus and fetal cortex. GDM-STZ treated with *Ganoderma lucidum*. SC (0.9% saline solution), GDM+S (Diabetic+saline solution 0.9%), GDM+Glb (Diabetic + 100 mg/kg/day of *Ganoderma lucidum* from gestation day 1 to 19), GDM+Gla (Diabetic + 100 mg/kg/day of *Ganoderma lucidum* from gestation day 9 to 19). Data are presented as mean ± SD (*n* = 8). (*) *p* < 0.05 in comparison to the SC group, One-way ANOVA, followed by Tukey–Kramer’s test. Note: H&E 40X photomicrography of a fetal encephalon (the yellow arrow indicates the cortex and the red arrow, the hippocampus).

Figure 6 presents the hippocampal and cortex measurements of the fetal brain. Only the GDM+SC group presented a decrease in hippocampal measurement when compared to SC (F = 3.80 and *p* < 0.05). In relation to cortex, no significant differences were observed (F = 2.28 and *p* > 0.05).

## 4. Discussion

DM is a chronic inflammatory disease in which a deficiency in insulin secretion or action results in insulin resistance and, consequently, hyperglycemia. The chronic increase in glucose damages various tissues and organs, especially the eyes, kidneys, nerves, heart, and blood vessels [28,29]. GDM is a type of diabetes related to pregnancy and it can increase the risk of fetal loss, complications at birth, congenital malformations, premature birth and a tendency to develop type 2 diabetes in the future [30].

In this sense, the Diabetes Association advocates fasting blood glucose testing early in the prenatal period to detect a possible increase in glycemia once the clinical signs of hyperglycemia may be confused with the initial symptoms of gestation. Hyperglycemia can cause maternal and fetal damage, threatening gestation if not controlled in time [31]. In a more significant screening, the oral glucose tolerance test (OGTT) is recommended for the period between 24 and 28 weeks of gestation for all pregnant women [32].

For understanding the GDM mechanisms and their consequences on a mother and conception, preclinical studies using animal models are essential to recognize maternal complications, congenital malformations, changes in placental physiology, and fetal development. The broader in vivo model for GDM is the induction of diabetes with STZ [33,34]. STZ is cytotoxic for beta-pancreatic cells, and it can override its function by increasing pro-apoptotic factors and accumulation of oxygen-reactive substances (ROS), ending up in cell death and diabetes [9,35]. The pancreas has both exocrine functions through pancreatic acini and endocrine function through the islets of Langerhans. Rats’ islets have a different architecture with a nucleus of β surrounded by other types of endocrine cells [36]. β-glucans from Gl can increase the factor that prevents apoptosis and also enhances the activity of antioxidant enzymes, reducing β-pancreatic cell lesions induced by STZ [37,38].

In our study, the OGTT evidenced the GDM-STZ establishment since all diabetic groups showed glycemia increased in all test times, simulating what happens in uncontrolled GDM. However, the Gl administration before and after STZ treatment was able to reduce the glycemic response to the glucose load. Maternal hyperglycemia is responsible for fetal hyperglycemia and it generates prolonged stimulation in fetal β-pancreatic cells. This stimulation leads to insulin depletion and fetal hypoinsulinemia, contributing to low fetal weight in mothers with severe diabetes [34]. Our results corroborated maternal hypoinsulinemia and hyperglycemia. *Ganoderma lucidum* was not able to revert the effects of STZ on maternal insulin levels.

The ALT and AST enzymes are important markers of liver function, and when they increase in blood, there are some visceral disorders, especially liver, renal and musculature disorders. The GDM-STZ rats usually show hepatic complications due to the disorganization in that tissue, such as fatty alterations, mitochondrial damage and vesiculations in the rough endoplasmic reticulum [39]. The biochemical profile of the GDM-STZ groups, in this study, presented a correlation between ALT and AST compatible with the STZ-induced lesions. On the other hand, groups exposed to Gl presented a reduction of these enzymes, suggesting a protective effect of this mushroom on liver cells.

In pregnancy, lipid metabolism changes to meet maternal and fetal energetic needs as gestation progresses; thus, a slight increase in total cholesterol, triglycerides and LDL-Chol is expected [40]. However, severe GDM-STZ shows a poor insulinotropic response to glucose load stimulation, resulting in hyperglycemia and exaggerated hypertriglyceridemia. On the other hand, maternal triglycerides do not cross the placental barrier, and the placenta is responsible for correlating maternal and fetus serum triglycerides [41]. LDL-Chol presents polyunsaturated fatty acids, able to induce lipid peroxidation, generating an imbalance between free radicals and antioxidants, and consequent oxidative stress [42]. *Ganoderma lucidum* was capable of reducing triglyceride levels when administered after fetus implantation. On the other hand, it did not alter total cholesterol levels. Concerning HDL-Chol, all the diabetic groups presented a reduction in values.

During pregnancy, increased levels of free radicals and lipid peroxides are expected [43]. Moreover, hyperglycemia during pregnancy generates ROS, which is responsible for oxidative stress in the intrauterine environment. Oxidative stress triggers congenital malformations and oxidative damage in fetal mitochondria and placental DNA in offspring [33,44].

Catalase, GPx and GSH are antioxidant biomarkers imbalanced in diabetes due to the metabolic syndrome. In the same way, lipid peroxidation tends to increase [45]. This evidence corroborated our findings in which the CAT and GPx activities decreased and TBARS increased in GDM+S. The oral administration of Gl to diabetic pregnant rats significantly reduced TBARS levels compared with diabetic saline, suggesting that mushrooms may improve the pathological condition of diabetes by inhibiting lipid peroxidation. Mushrooms contain a wide variety of bioactive ingredients that encourage their application in disease prevention and human health maintenance [46]. Moreover, Gl administered after fetus implantation increased CAT activity.

Comparatively, Gl reduced the damage induced by oxidative stress against myocardial infarction by evaluating its antioxidant function through the dosages of GSH and malondialdehyde [47]. An increase in CAT and GPx activities was found in diabetic rats treated with *Ganoderma lucidum* when compared to the diabetic control group [48]. Pan and colleagues highlighted the antioxidant potential of *Ganoderma lucidum* in diabetic nephropathy in rats, as it reduced lipid peroxidation and increased CAT activity [45].

Regarding embryofetal development, hyperglycemia in GDM is responsible for causing substantial deleterious effects in the fetal brain. Hyperglycemia decreases the number of neuronal cells changing the thickness of the white matter and gray matter. Besides, hyperglycemia inhibits retinoic acid which is responsible for the differentiation of the cortical neurons and possesses antioxidant activity in the central nervous system [49]. In our study, there were no marked abnormal histological changes in the brains of GDM-fetuses.

Moreover, no changes were observed in the fetus kidneys. In a chronic kidney disease (CKD) study with children, Hsu and colleagues concluded that maternal overweight and low fetal birth weight related to GDM were responsible for increasing cases of CKD in infants [50].

This study had two main limitations. One of them was related to the amount of blood collected from the rats, which was not enough to implement new analyses or replicate the performed analyses. The other one was the STZ-model in pregnant rats. There is no specific and standardized STZ dose to induce gestational-DM. STZ can induce severe diabetes, and often, in pregnancy, the diabetes is moderate. The STZ-model does not distinguish between Type 1 and Type 2 DM. The pregnant rats were still diabetic because nutraceuticals cannot replace diabetic therapy. However, this study overcame these limitations, showing protective effects on fetuses and regulating diabetes secondary complications.

## 5. Conclusions

β-glucans and phenolic compounds from *Ganoderma lucidum* showed important protective activity in gestational diabetes induced by streptozotocin, strengthening its nutritional and medicinal potentiality in diabetes. Although pregnant rats were still diabetic at the end of the study, *Ganoderma lucidum* was prospective as a nutritional therapeutic agent for diabetes complications, mainly by regulating glucose metabolism and preventing lipid oxidation, without adverse effects.

## Figures and Tables

**Table 1:** Reproductive Capacity of GDM-STZ rats treated with *Ganoderma lucidum*.

Groups	Uterus Weight (g)	Ovary Weight (g)	Offspring Vitality	Post-Implantation Loss (%)
SC	44.48 ± 13.83	0.142 ± 0.015	9.75 ± 1.83	4.6
GDM+S	35.40 ± 12.49 *	0.112 ± 0.019 *	8.16 ± 1.53	16 *
GDM+Glb	43.18 ± 2.95	0.117 ± 0.017	11.0 ± 1.58	13.8 *
GDM+Gla	48.52 ± 3.89	0.104 ± 0.020	11.83 ± 0.69	14.1 *
	(F = 1.159, *p* = 0.03539)	(F = 8.561, *p* < 0.0001)	(F = 1.608, *p* = 0.2269)	(*p* = 0.0367)

SC (0.9% saline solution), GDM+S (Diabetic+saline solution 0.9%), GDM+Glb (Diabetic + 100 mg/kg/day of *Ganoderma lucidum* from gestation day 1 to 19), GDM+Gla (Diabetic + 100 mg/kg/day of *Ganoderma lucidum* from gestation day 9 to 19). Data are presented as mean ± SD (*n* = 6). (*) *p* < 0.05 in comparison to the SC group, one-way ANOVA, followed by Tukey–Kramer’s.

## Data Availability

Data is contained within the article and supplementary material.

## Supplementary Materials

The following are available online at https://www.mdpi.com/article/10.3390/antiox11061035/s1, Figure S1 Fingerprint composition of the Ganoderma lucidum (Gl) mushroom methanolic extract by Chromatogram High Performance Liquid Chromatography. In (A) general chromatogram; (B) chromatogram with retention times. and Figure S2. Fingerprint composition of the Ganoderma lucidum (Gl) mushroom chloroform extract by Chromatogram High Performance Liquid Chromatography. In (A) general chromatogram; (B) chromatogram with retention times.
