# Supplementary material for: Ganoderma lucidum Modulates Glucose, Lipid Peroxidation and Hepatic Metabolism in Streptozotocin-Induced Diabetic Pregnant Rats
Source: Antioxidants (Basel). 2022 May 24;11(6):1035. doi: 10.3390/antiox11061035 (PMC9219838; doi:10.3390/antiox11061035)

Supplementary Material

Supplementary Figure S1. Fingerprint composition of the *Ganoderma lucidum* (Gl) mushroom methanolic extract by Chromatogram High Performance Liquid Chromatography. In (A) general chromatogram; (B) chromatogram with retention times.

A

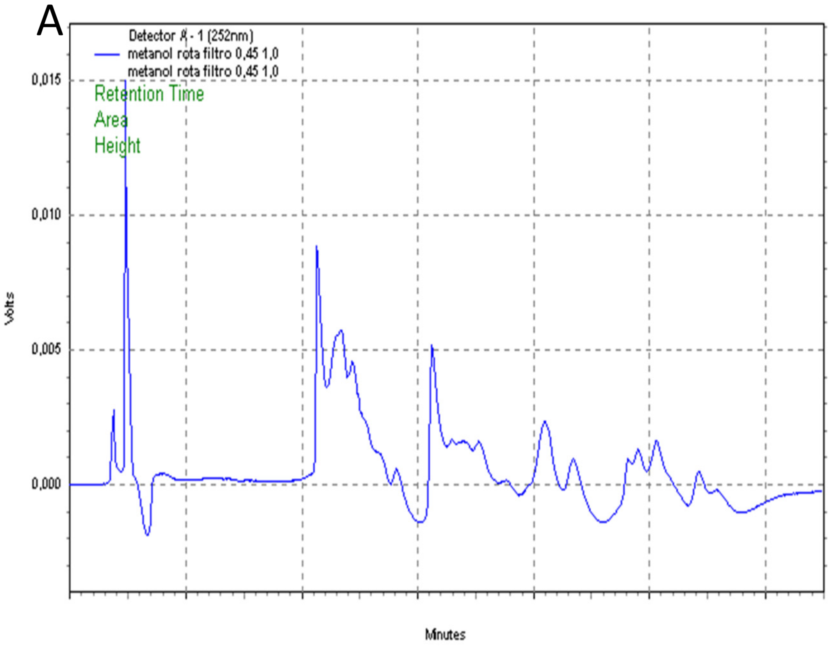

B

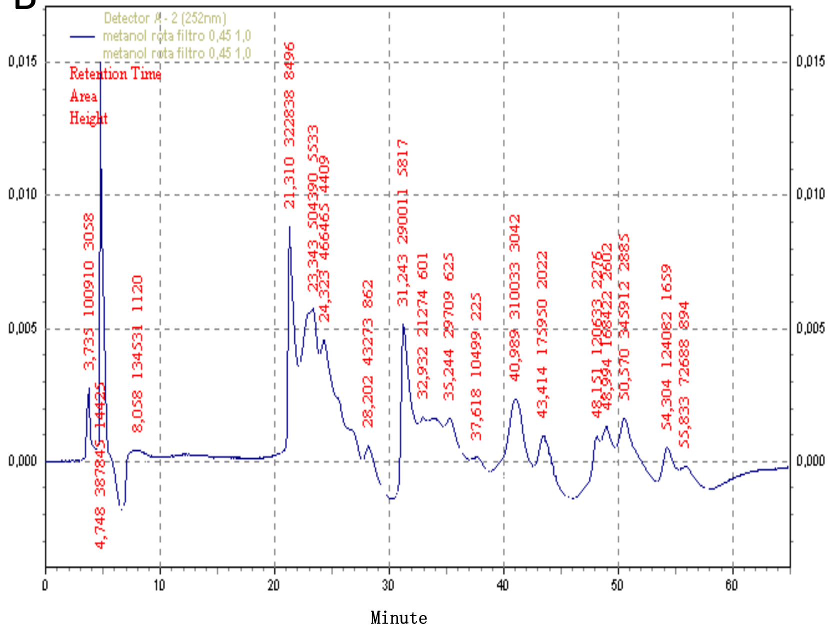

Supplementary Figure S2. Fingerprint composition of the *Ganoderma lucidum* (Gl) mushroom chloroform extract by Chromatogram High Performance Liquid Chromatography. In (A) general chromatogram; (B) chromatogram with retention times.

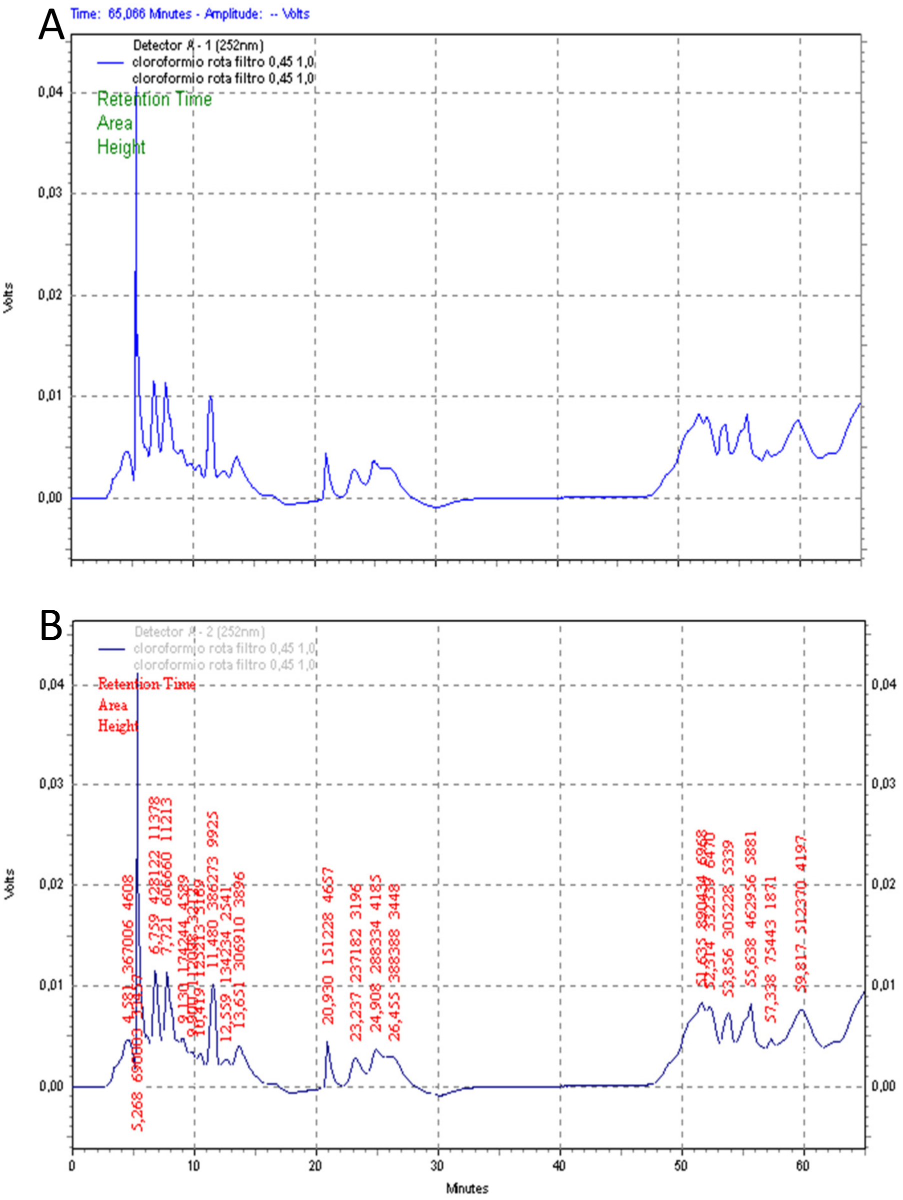

Supplement: Supplementary file 1 [file antioxidants-11-01035-s001.zip › antioxidants-1651059-supplementary.pdf]
